# MicroRNA profiling of novel African American and Caucasian Prostate Cancer cell lines reveals a reciprocal regulatory relationship of *miR-152* and DNA methyltranferase 1

**DOI:** 10.18632/oncotarget.1953

**Published:** 2014-05-08

**Authors:** Shaniece C. Theodore, Melissa Davis, Fu Zhao, Honghe Wang, Dongquan Chen, Johng Rhim, Windy Dean-Colomb, Timothy Turner, Weidong Ji, Guohua Zeng, William Grizzle, Clayton Yates

**Affiliations:** ^1^ Department of Biology and Center for Cancer Research, Tuskegee University, Tuskegee, AL; ^2^ Center for Prostate Disease Research, Department of Surgery, Uniformed Services University of the Health Sciences, Bethesda, MD; ^3^ Department of Genetics, University of Georgia, Athens, GA; ^4^ Department of Pathology, University of Alabama at Birmingham School of Medicine, Birmingham, AL; ^5^ Department of Urology, Minimally Invasive Surgery Center, The First Affiliated Hospital of Guangzhou Medical College, Guangdong Provincial Key Laboratory of Urology, 1 Kangda Road, Guangzhou 510230, China; ^6^ Division of Preventive Medicine, University of Alabama at Birmingham School of Medicine, Birmingham, AL; ^7^ Department of Oncologic Sciences, University of South Alabama Mitchell Cancer Institute, Mobile, AL

**Keywords:** miRNA, DNA methylation, African American, prostate cancer

## Abstract

miRNA expression in African American compared to Caucasian PCa patients has not been widely explored. Herein, we probed the miRNA expression profile of novel AA and CA derived prostate cancer cell lines. We found a unique miRNA signature associated with AA cell lines, independent of tumor status. Evaluation of the most differentially expressed miRNAs showed that miR-132, miR-367b, miR-410, and miR-152 were decreased in more aggressive cells, and this was reversed after treatment of the cells with 5-aza-2′-deoxycytidine. Sequencing of the miR-152 promoter confirmed that it was highly methylated. Ectopic expression of miR-152 resulted in decreased growth, migration, and invasion. Informatics analysis of a large patient cohort showed that decreased miR-152 expression correlated with increased metastasis and a decrease in biochemical recurrence free survival. Analysis of 39 prostate cancer tissues with matched controls (20 AA and 19 CA), showed that 50% of AA patients had statistically significant lower miR-152 expression compared to only 35% of CA patients. Ectopic expression of miR-152 in LNCaP, PC-3, and MDA-PCa-2b cells down-regulated DNA (cytosine-5)-methyltransferase 1 (DNMT1) through direct binding in the DNMT1 3'UTR. There appeared to be a reciprocal regulatory relationship of miR-152/DNMT1 expression, as cells treated with siRNA DNMT1 caused miR-152 to be re-expressed in all cell lines. In summary, these results demonstrate that epigenetic regulation of miR-152/DNMT1 may play an important role in multiple events that contribute to the aggressiveness of PCa tumors, with an emphasis on AA PCa patients.

## INTRODUCTION

The most commonly diagnosed type of cancer among men in the US is prostate cancer (PCa), which accounts for 29% (241,740) of all new cancer cases. Although the number of new cases of PCa has decreased in recent years, there are still racial and ethnic differences in PCa epidemiology. African-Americans (AAs) have the world's highest incidence of PCa and more than twofold higher mortality rate compared with Caucasian Americans (CAs) [1]. Overall, AA patients are younger and have higher Gleason scores, PSA levels, and incidence of palpable disease [2]. Various factors have been associated with the more aggressive prostate tumors. For example, differential gene expression in AA patients contributes to aggressive disease [3-7], and epigenetic mechanisms, such as DNA methylation, result in the loss of key regulatory genes [8, 9]. This is particularly evident for AA patients where hypermethylation of genes in normal or pre-malignant areas are thought to predispose to malignancy [10, 11]. However, the underlying mechanism of these acquired methylation patterns is poorly understood.

MicroRNAs (miRNAs) are small RNA molecules consisting of 19–23 nucleotides that regulate various biological processes. More than 60% of protein-coding genes may be targeted by miRNAs [12], mainly through translational repression and degradation of target mRNAs. An expanding body of evidence supports a role for miRNAs in disease progression and the potential for epigenetic mechanisms, such as DNA methylation, to regulate miRNA expression [13, 14]. Recently, DNA methylation of proximal CpG islands in miRNA promoters was described as a method for decreased expression in various cancers, including PCas [15-18]. Although miRNAs are expressed differently in healthy tissues and cancers [19] and in localized and advanced tumors [20], little is known about racial differences in miRNA expression. Identification of unique miRNAs and mRNA-associated targets will begin to clarify the specific events involved in the progression of PCas in AAs.

To address this question, our laboratory has established non-malignant and malignant cell lines derived from AA PCas that replicate many of the clinical features of the original PCas [21, 22]. Thus, utilizing these cell lines and commonly available PCa cell lines, we explored the possibility of race-related differences in miRNA expression. Herein, we report that AA cell lines have a distinct miRNA signature, independent of tumor status. Evaluation of the miRNAs most differentially expressed demonstrated that miR-132, miR-367b, miR-410, and miR-152 were decreased in more aggressive cells and that this expression was reversed after treatment of the cells with 5-aza-2′-deoxycytidine (5-aza-2'd). Bisulfite conversion and sequencing of the promoter showed that miR-152 was highly methylated in LNCaP and PC-3 cells. Ectopic expression of miR-152 resulted in decreased cell proliferation, migration, and invasion. In a panel of 39 PCa tumors with adjacent matched controls (20 AA and 19 CA), 50% of AA patients and 35% of CA patients demonstrated statistically significant lower miR-152 expression compared to adjacent controls. Ectopic expression of miR-152 down-regulated DNA(cytosine-5)-methyltransferase 1 (DNMT1) through direct binding in the DNMT1 3'UTR. This appeared to be an inverse relationship, as cells treated with DNMT1 siRNA re-expressed miR-152. Finally, as determined with a large patient cohort, loss of miR-152 was related to poor clinical outcomes including decreased biochemical recurrent free survival.

## RESULTS

### miRNA Profile of Panel of AA and CA Cell Lines

**Fig. 1:**
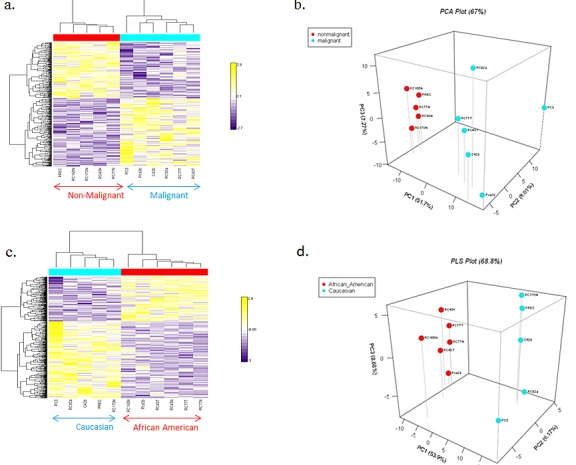
Heat Map of miRNA microarray (a) Hierarchical clustering analyses of miRNAs (left) grouped by non-malignant and malignant status: non-malignant cell lines (red block), malignant cell lines (blue block). (b) PLS plot of AA and CA prostate cell lines grouped by malignancy. (c) Hierarchical clustering analyses of miRNAs (left) grouped by race. AA cell lines (red block), CA cell lines (blue block). miRNAs are ordered according to their cluster determined by p-values using the Kruskal-Walis test. (d) PLS plot of AA and CA prostate cell lines grouped by race.

To determine the miRNA expression pattern in our panel of AA and CA prostate cell lines, the expression of 662 miRNAs was analyzed utilizing Asuragen Affymetrix Gene Chips. To validate the malignant and non-malignant status of this cell line panel, we first preformed hierarchical clustering (Figure 1a) and a PLS plot (Figure 1b), using reported non-malignant and malignant status as the grouping criteria. Both analysis demonstrated distinct expression patterns in the malignant and non-malignant cell lines, with 21 differentially expressed miRNAs between the groups (Supplemental Table 1 (p<0.00001). To further determine race related miRNAs, we performed a separate hierarchical clustering analysis on the same data set utilizing racial background as the grouping criteria. Distinct hierarchical clustering of miRNAs was evident between the AA and CA cell lines (Figure 1c), regardless of malignancy status. A PLS plot reflected this pattern, with distinct variations in the racial profiles of the AA and CA cell lines (Figure 1d). In this variation, there were 47 miRNAs differentially expressed by race (Supplemental Table 2). To confirm these 47 miRNAs are race related, we interrogated a microarray dataset that consisted of primary tumor cells isolated from 5 AA and 4 CA patients (Supplemental Table 3). These race related miRNA probes were able to differentiate patients relevant to race using PLS plot test (Supplemental Figure 1).

To determine if these miRNA's have a relationship to prostate cancer progression, we took the most significantly differentially expressed race related miRNA's (p<0.00001) (Table 1), and performed qRT-PCR validation within our prostate cancer cell line progression model. Of the 11 miRNAs, we confirmed the 5 most significant by qRT-PCR in non-malignant and selected aggressive malignant cell lines (Supplementary Figure 2). With the exception of miR-363, which did not exhibit a clear expression pattern, miR-132, miR-376b, miR-410 and miR-152 exhibited a decreased expression profile as the metastatic capability of cell lines increased. Thus, we chose these miRNAs for further analysis.

**Table 1:** Hierarchical clustering of race related miRNAs

miRNA	(Adjusted) p-value ↑
hsa-miR-363	3.43e-05
hsa-miR-132	0.000354
hsa-miR-376b	0.00188
hsa-miR-410	0.00291
hsa-miR-152	0.00471
hsa-miR-189	0.00498
hsa-miR110	0.00514
hsa-miR-27b	0.00657
hsa-miR-519c	0.0071
hsa-miR-520h	0.00774
hsa-miR-27a	0.00837

**miRNAs separated by race are listed based on p-value

### Demethylation Treatment Reverses miRNA Expression of AA-Associated miRNAs

**Fig 2:**
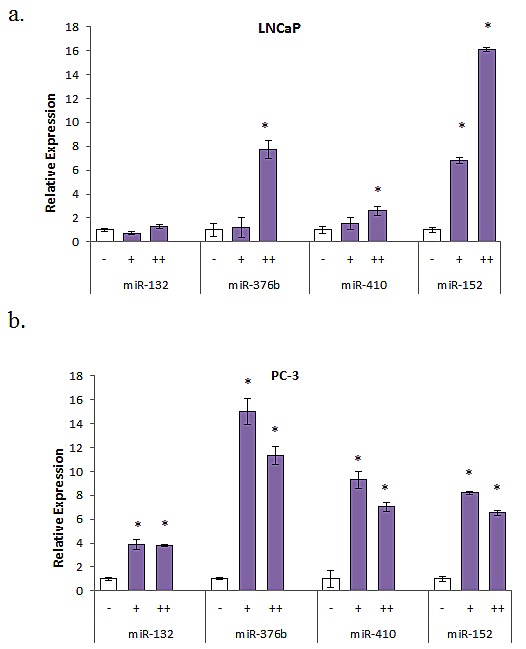
Multiple miRNA expression pattern after treatment with demethylation agent (a) LNCaP cells were treated with 5 μM 5-aza-2'd for 4 days alone (+) or with 100 nM TSA for 24 hr (++). Results shown are representative of three independent experiments ± s.e.*P<0.05. (b) PC-3 cells were treated with 5 μM 5-aza-2'd for 4 days alone (+) or with 100 nM TSA for 24 hours (++). Results shown are representative of three independent experiments ± s.e. *P<0.05.

Previous reports and *in silico* analyses have demonstrated that a majority of the miRNAs we found associated with race contain CpG islands within the promoter regions upstream of the start site (Supplemental Figure 3). To determine if hypermethylation is associated with decreased expression of these miRNAs, LNCaP and PC-3 cells were treated with 5 μM 5-aza-2'd alone for three days or in combination with 100 nM TSA for 24 hr. Re-expression of multiple miRNAs was evident in both LNCaP and PC-3 cells after 5-aza-2'd treatment. However, miR-376b and miR-152 showed the most substantial increases in both cell lines (Figure 2).

**Fig 3:**
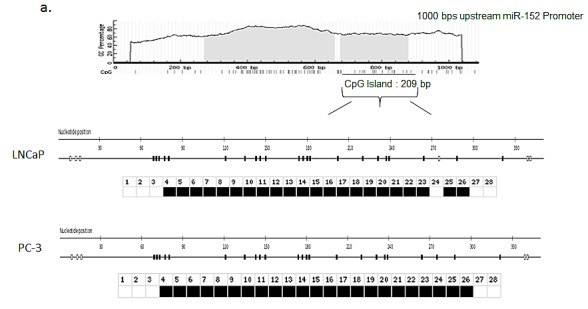
Bisulfite Sequencing of CpG islands in the miR-152 promoter (a) Schematic map of the CpG islands in the *miR-152* promoter using UCSC Genome Browser. CpG islands are indicated as vertical lines on map. (b) Bisulfite modification was performed on LNCaP and PC-3 cells, and DNA was sequenced to determine the methylation status of the promoter region. Bisulfite sequencing was analyzed by BDPC software (*see Methods*). Open square; unmethylated CpG; closed square, methylated CpG.

Since miR-152 contained the greatest percentage of methylated CG sequences and demonstrated the most consistent increases after 5-aza-2'd treatment, we focused on this miRNA. To determine the methylation status of the miR-152 promoter region, we extracted DNA from LNCaP and PC-3 cell lines and performed sodium bisulfite modification prior to sequencing. To confirm these results, sodium bisulfite-converted DNA was subjected to capillary electrophoresis and analyzed utilizing the online Bisulfite Sequencing DNA Methylation Analysis (BISMA) sequencing program. The results indicated that DNA of both LNCaP and PC-3 cell lines was 100% methylated at 1000 base pairs upstream from the promoter region (Figure 3). Thus, these results suggest that in malignant PCa cell lines, miR-152 is inactivated through hypermethylation.

### miR-152 Expression Correlates with Clinical and Pathological Variables

**Fig 4:**
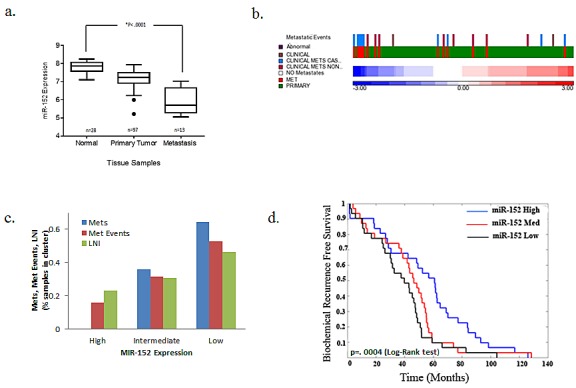
Low miR-152 expression correlation with PCa metastasis Taylor et al data set samples were sorted by average miR-152 expression and the mean from two probes was normalized with z-score. (a-d) miR-152 expression correlates with metastases, invasion events, and recurrence-free survival in 138 PCa samples (97 primary tumors and 13 metastases). (a) miR-152 levels decrease monotonically from normal, primary tumor, and metastasis, *P* < 0.0001. (b) Intensity in heatmap, with red corresponding to high and blue to low expression, with common event that occur in PCa metastasis. (c) Number of metastatic events separated by high, intermediate, and low miR-152 expression. (d) Low miR-152 expression correlated with decrease in probability of biochemical recurrence free survival p=0.0004 (log-rank test).

The expression of miR-152 in 28 normal cell lines, 97 primary tumors, and 13 metastases was analyzed using the Taylor et al. GSE21032 data set available on the GEO website (http://www.ncbi.nlm.nih.gov/geo/). Primary and metastatic tumors had lower levels of miR-152 relative to normal samples (Figure 4a), which correlated with the higher incidence of metastatic samples, metastatic events, and lymph node invasion (Figures 4b and c). Tumors with low miR-152 levels also had reduced biochemical recurrence-free survival (Figure 4c). Together, this describes a consistent picture of low miR-152 levels associated with PCa metastasis and recurrence.

**Fig 5:**
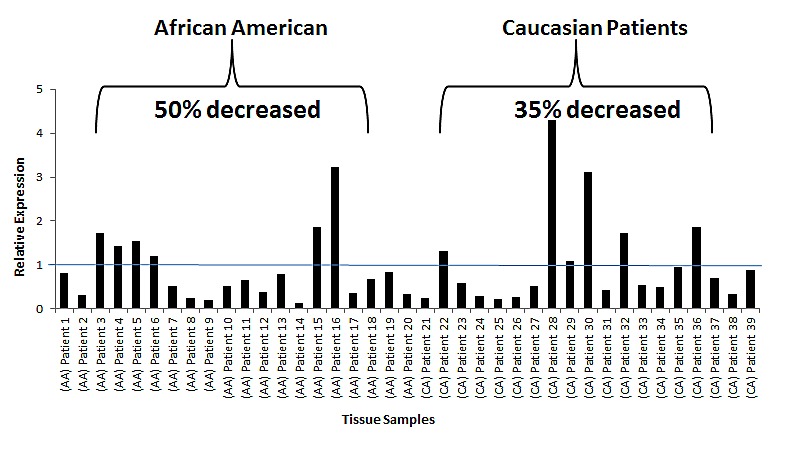
miR-152 expression in AA and CA matched normal tumor cohort (a) qRT-PCR of miR-152 expression in normal-tumor paired prostate tissue samples. All samples were normalized to the adjacent normal samples. All values under value one represent down-regulation of miR-152. (b) Plot demonstrating the individual miR-152 expression values in AA and CA patients: Note: 50% of the individual AA patients had significant lower miR-152 expression compared to only 35% of CA patients. p-values (Benjamini-Hochberg corrected for multiple tests).

These results prompted us to determine miR-152 expression in our patient cohort of AAs and CAs, hypothesizing that tumors from AAs would have lower miR-152 than those from CA patients. Patients were selected based on cancers of higher total Gleason score (≥6) and/or pathological stage (pT2), and positive for perineural and/or vascular invasion, as these pathological characteristics correlate positively with tumor aggressiveness and metastasis. AA patients had a lower median age relative to similarly staged CA patients, which was also associated with lower miR-152 levels measured by qRT-PCR (Table 2, p<.001). Analysis of miR-152 expression in individual tumors compared to matching adjacent normal controls showed a statistically significant decrease in miR-152 expression in 50% of the AA patients compared to only 35% of CA patients (Figure 5a, b Supplemental table 4).

**Table 2:** Clinical Characteristics of Paired Primary Tumor Prostate Samples

Ethnicity	African American	Caucasian	p†
Samples	20 (51.3%)	19 (48.7%)	
Age			
Median	57.5	64	
Mean	57.3	60	
Standard deviation	6.68	7.3	
Min - Max	47-69	48-70	
Initial Clinical Stage			
T1	NA	NA	
T2	16 (80%)	10(52.6%)	
T3	4 (20%)	8 (42.1%)	
NS	NA	1 (5.3%)	
Gleason Score			
6	8 (40%)	8 (42.1%)	
7	12 (60%)	10 (52.6%)	
8	NA	1 (5.26)	
miR-152 Expression			
Average All Samples ±SD	1.6±	2.6±	p<.001*

†P value with * is deemed significant utilizing F- test.

NA- none

SD- Standard Deviation

### Restoring miR-152 Expression Decreases Cell Growth

**Fig 6:**
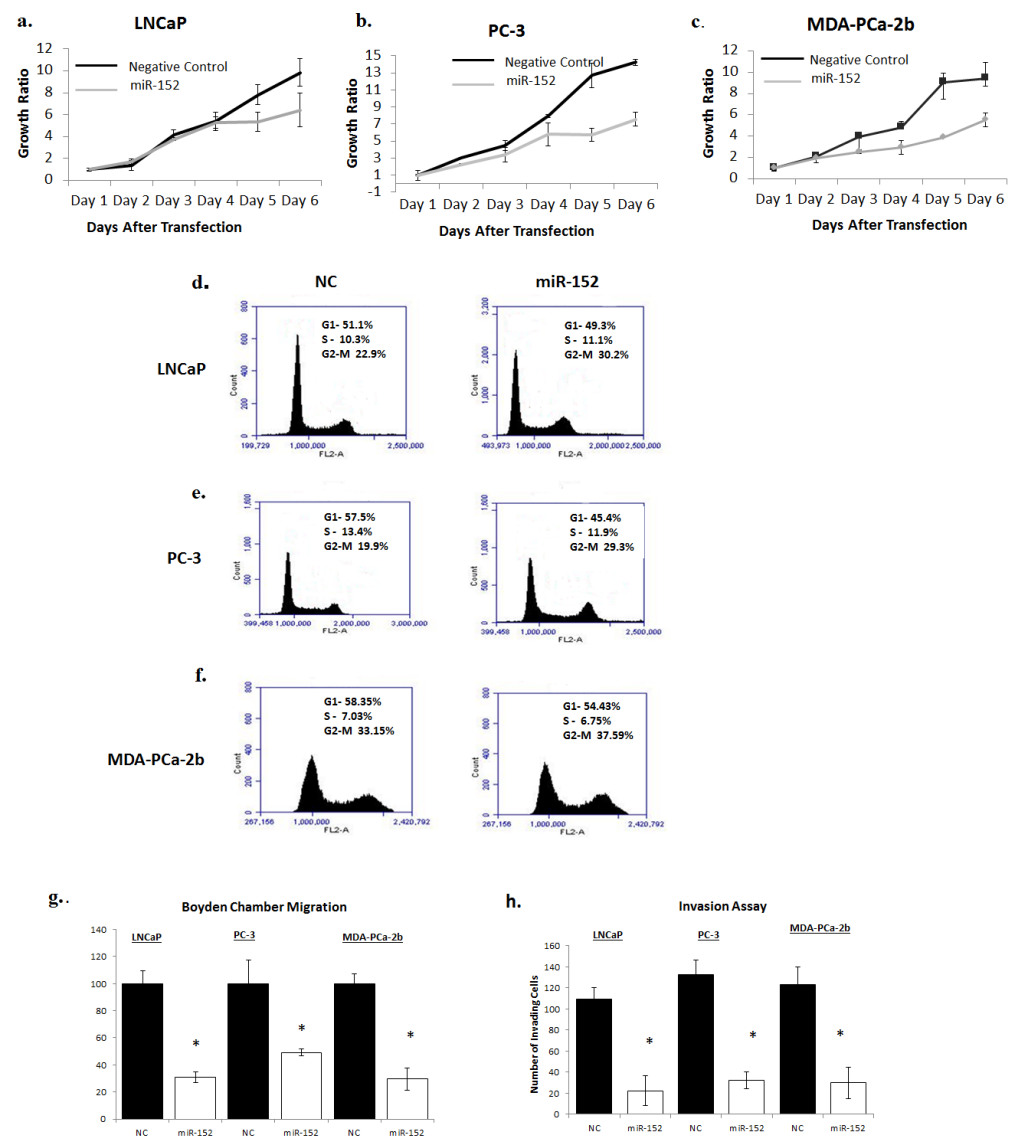
Restoring miR-152 expression decreases cell proliferation, migration, and invasion (a) Proliferation of LNCaP, (b) PC-3, (c) and MDA-PCa2b cells that were transfected with either 30 nM of miR-152 mimic or miR-NC (negative control) was measured by MTT for 6 days. Results shown are representative of three independent experiments averaged and normalized to Day 1 ± s.e. *p<0.05. (d) LNCaP, (e) PC-3, (f) and MDA-PCa-2b cells were analyzed by flow cytometry after 3 days of miR-152 mimic transfection (30 nM). miR-152 caused G2-M arrest in cell cycle progression in PCa cell lines. Results shown are representative of three independent experiments. (g) Relative cell migration of LNCaP PC-3, and MDA-PCa-2b cells was measured utilizing Boyden migration chambers. Results shown are representative of two individual experiments performed in triplicate. (h) LNCaP, PC-3, and MDA-PCa-2b cells transfected with miR-152 mimic showed a decrease in the number of cells invading through a layer of Matrigel relative to cells treated with miR-NC (negative control). All data presented are the means of three independent experiments ± s.e. *P<0.05.

The clinical relevance of miR-152 in PCa prompted us to determine if loss of expression had a biological or functional role in promoting metastatic tumors. After optimizing the concentration of miR-152 mimics that restored miR-152, miR-152 was transfected into LNCaP, PC-3, and MDA-PCa-2b cells. As determined by MTT assays, ectopic expression of miR-152 inhibited cell proliferation after 3 days, and this continued to Day 6 (Figure 6 a,b,c). Since the inhibition of cell proliferation began at 72 hr, miR-152 transfected cells were next assayed after 72 hr by flow cytometry to determine if there was an effect on cell cycle progression. miR-152 treatment caused cells to accumulate at the G2-M phase (LNCaP NC 22.90% compared to miR-152-transfected cells 30.2%; PC-3 NC 19.9% vs miR-152 transfected cells 29.3%; MDA-PCa-2b NC 33.15 vs miR-152 transfected cells 37.59%) (Figure 6 d, e, f). In addition to reduced cell proliferation, miR-152 transfected LNCaP, PC-3, and MDA-PCa-2b cells demonstrated a decrease in cell migration (Figure 6g) and invasion (Figure 6h).

**Fig 7:**
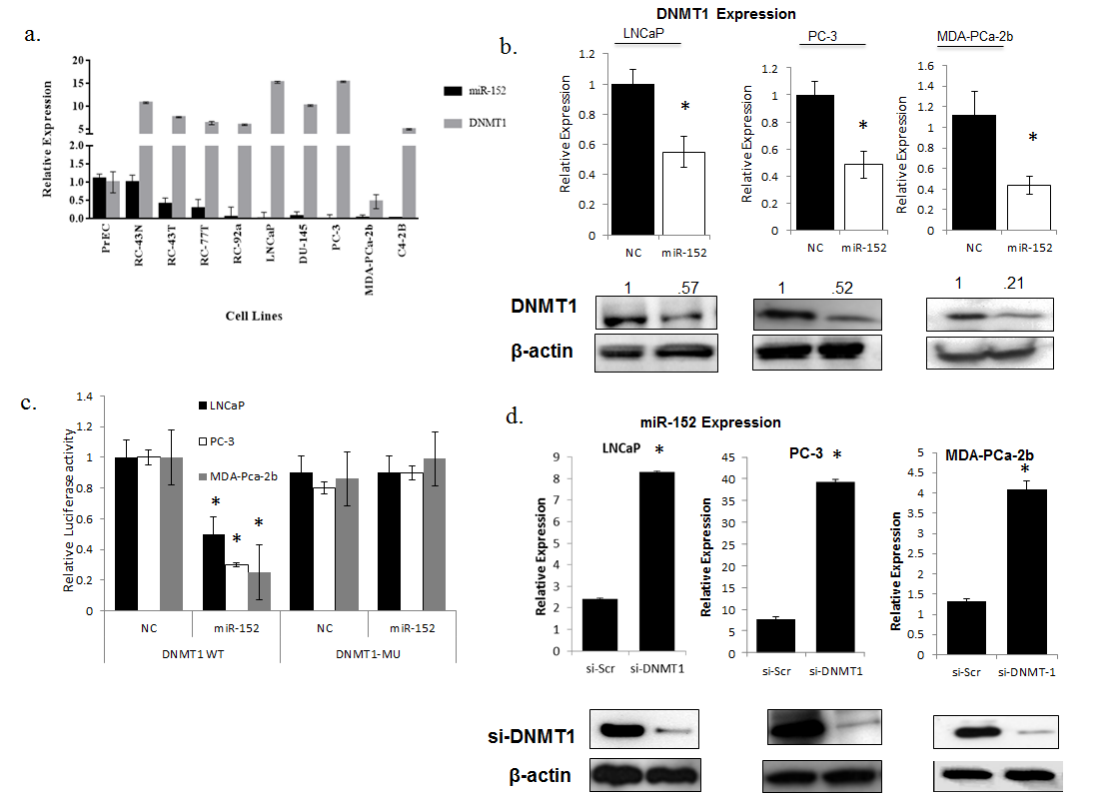
Comparison of miR-152 and DNMT-1 relationship in PCa cell lines (a) Relative miRNA and mRNA expressions of miR-152 and DNMT1 were determined by qRT-PCR in a panel of prostate cell lines with increasing aggressiveness. (b) miR-152 or scrambled oligonucleotides as NC (negative control) treatment of LNCaP, PC-3, MDA-PCa-2b cells resulted in decreases of DNMT1 at both RNA and protein levels. (c) Dual-luciferase assays were performed for LNCaP, PC-3, and MDA-PCa-2b cells co-transfected with the firefly luciferase constructs containing the DNMT1 wild-type or Mu 3′-UTR and miR-152 mimics or (NC) negative control. (d) siRNA-DNMT1 treatment resulted in increases in miR-152 expression in LNCaP, PC-3, and MDA-PCa-2b cells, as determined by qRT-PCR. DNMT1 expression was analyzed by immunoblots utilizing anti-DNMT1 antibody for both LNCaP, PC-3, a nd MDA-PCa-2b cells after siR-DNMT1 treatment. For all qRT-PCR experiments, expression was normalized to RNU48 (miRNA) and GAPDH (mRNA) controls. All data presented are the means of three independent experiments ± s.e. *P<0.05.

Previously, miR-152 was demonstrated to target DNMT1 expression in endometrial tumors [23]. Since we observed that numerous miRNAs in our panel were influenced by methylation, and the methyltransferase DNMT1 is a regulator of DNA hypermethylation in various tumor types, we sought to explore the miR-152/DNMT1 relationship in PCas. First, the relative expressions of DNMT1 and miR-152 were examined in our panel of cell lines. As expected, qRT-PCR showed that DNMT1 expression was elevated in the more aggressive cell lines, and this correlated with decreased expression of miR-152 (Figure7a).

DNMT1 expression was then examined in LNCaP, PC-3, MDA-PCa-2b cells transfected with miR-152. Both types of cells showed statistically significant decreases in DNMT1 expression at the RNA and protein levels (Figure 7b). This relationship is possibly direct, as miR-152 has putative binding sites in position 45-54 of the DNMT1 3' UTR. To confirm this, we utilized a previously established DNMT1 3′-UTR luciferase reporter system [24] containing the miR-152-binding sites (DNMT1 wild-type 3′- UTR) or mutating these sites (DNMT1 Mu 3′-UTR) that contain the putative miR-152 binding sites. All miR-152 transfected cells showed decreased DNMT1 wild-type 3′-UTR luciferase activity, whereas DNMT1 Mu 3′-UTR luciferase activity was not affected (Figure 7c). Since we demonstrated that loss of miR-152 occurs through promoter methylation, we asked if there was a reciprocal relationship between miR-152 and DNMT1. Treatment of LNCaP, PC-3, and MDA-PCa-2b cells with DNMT1 siRNA caused statistically significant increases in miR-152 expression (Figure 7d).

To examine the possible broader influence that loss of miR-152 has in PCas, we queried the TargetScan *in silico* database to determine additional gene targets that could be regulated by miR-152. Of the top genes, Rictor, TGF-β, SOS1, ABCD3, SMAD4, SOX2, E2F1, and Dicer were predicted. To determine their influence, a custom gene array of these genes was designed (Supplemental Table 5), and expression levels in LNCaP, PC-3, and MDA-PCa-2b cells that were transfected with miR-152 were assayed. Although, the effect of ectopic miR-152 on gene expression varied among the cell lines, we observed consistent decrease expression in Rictor, TGF-β, and SOS1. Interestingly, in the African American derived MDA-PCa-2b, we observed significant decreases in ABCD3 and SOS1, which have been previously reported to be associated with African American prostate tumors [4, 5]. Together, these results indicate that miR-152 regulates epigenetic events that promote tumorigenesis.

## DISCUSSION

Numerous studies have now reported gene differences AA and CA prostate tumors [4, 6, 7, 25, 26]. As the field of health disparities is in its infancy, these reports provide the first evidence of population-based genetic contributions to aggressive disease. However, the regulation of these genes is still elusive. To address this question, without influence of non-epithelial stromal cells typically present in tissues, we utilized AA- and CA-derived cell lines derived from benign and primary tumors, along with well-established cell lines derived from metastatic PCas. Our findings derived by miRNA microarray analysis revealed a distinct miRNA expression pattern in AA-derived cell lines. qRT-PCR validation of these miRNAs showed that the five most significant miRNAs were decreased in metastatic cell lines, suggesting a role for these molecules in tumorigenesis. Since previous reports have demonstrated inheritable epigenetic changes in AA patients, we sought to determine if decreased expression was the result of DNA methylation. *In silico* analysis revealed that these miRNAs have dense CG regions in the promoter. Further evidence that methylation of CpG islands causes silencing is that each of the miRNAs, albeit to varying degrees, is re-expressed after treatment with 5-aza-2'd or TSA. To our knowledge, this is the first report that demonstrates a methylated miRNA profile that can be associated with the progression of AA prostate tumors.

Of the miRNAs influenced by 5-aza-2'd, several have been found in other tumor types, including PCa. For example, miR-132, which targets both heparin-binding epidermal growth factor and TALIN2, is silenced by hypermethylation [15]. Of the miRNAs we assayed, miR-152 demonstrated the most significant reversal of expression, an effect consistent for both LNCaP and PC-3 cell lines. Previous reports regarding endometrial cancer [23], gastrointestinal cancer [27], and ovarian cancer [28, 29], and a report relating to PCa [30], published during the preparation of this manuscript, show that miR-152 levels are decreased in more advanced tumors. Analysis of patient data from the Taylor et al. study, deposited in the NIH-sponsored Geo database, confirms that miR-152 expression is low in the more advanced primary tumors, with the lowest expression in metastatic samples. However our analysis provides evidence that low miR-152 levels decreases probability of biochemical recurrence free survival in patients. qRT-PCR to measure normal-tumor expression ratios confirmed these findings, with 67% of patients displaying lower miR-152 expression. However, the most relevant observation is that AA patients display decreased expression of miR-152 in both uninvolved and paired tumors compared to CA patients of similar age, stage, and Gleason grade. To determine the mechanism for decreased miR-152, sodium bisulfite modification and sequencing of miR-152 promoter in aggressive PCa cell lines were performed. Both LNCaP and PC-3 cells showed 100% methylation status, highlighting that, in aggressive tumors, hypermethylation of the miR-152 promoter is a mode of silencing miR-152.

miR-152 directly targets the 3′ UTR of DNMT1 [23]. Across our cell line panel, DNMT1 and miR-152 showed an inverse relationship in expression. Although the miR-152/DNMT1 inverse expression relationship was significant in MDA-PCa-2b cells, both the LNCaP and PC-3 cells showed much greater miR-152/DNMT1 expression differences. However in all three cell lines, we confirmed that forced expression of miR-152 results in decreased expression of DNMT1 at both the RNA and protein levels. We also confirmed in LNCaP, PC-3, and MDA-PCa-2b cells decreased expression of several candidate targets that have been implicated in AA prostate tumors. SOS-1, which is a regulator of EGFR expression and downstream signaling, is increased in AA PCas, and most significantly decreased in the AA derived MDA-PCa-2b cells [5]. Although Rictor, a subunit of the mTORC2 complex, has not been directly implicated in AA PCas, the phosphatidylinositol-3-kinase/AKT (PI3K/AKT) pathway, which is a regulator of the mTORC2 complex, has been associated with AA PCas [25]. Whereas TGF-α is a direct target of miR-152 in PCa cell lines [30], we observed decreased TGF-β mRNA expression after forced miR-152 expression in all three cell lines, which further suggests that loss of miR-152 promotes increased aggressiveness through multiple signaling pathways. Previously, we have demonstrated that ABCD3 expression is associated with African American Prostate tumors [4.] Interestingly, we observed decreased ABCD3 expression in the MDA-PCa-2b cells after miR-152 ectopic expression, that was not observed in LNCaP or PC-3 cells. Because DNMT1 has been characterized as one of the main enzymes responsible for maintenance of global methylation patterns in tumor-related genes [31, 32], we investigated the role of DNMT1 on miR-152. We found that depletion of DNMT1 through siRNA resulted in increased miR-152 in both LNCaP, PC-3, and MDA-PCa-2b cell lines, highlighting that the effect is independent of androgen sensitivity. However, there appears to be a feed-forward loop where either loss of miR-152 and/or increased DNMT1 maintains methylation patterns, and hence miR-152 expression. miR-152 is gaining interest as a factor in various tumor types; a recent report concerning nickel sulfide-transformed human bronchial epithelial (16HBE) cells demonstrated that the miRNA-152/DNMT1 relationship develops early in transformed cells [24]. Treatment of cells with 5-aza-2'-d or depletion of DNMT1 led to increased miR-152 expression by reversal of promoter hypermethylation. These findings are similar to those presented here, however the available data relating to PCas suggest that the relationship between loss of miR-152 and increased DNMT1 occurs during the progression to advanced tumor status.

Our finding of a regulatory mechanism that maintains methylation patterns has implications for AA PCa patients. The fact that miR-152 expression is lower in non-malignant tissues from AA patients compared to CA non-malignant tissues suggests that miR-152 expression is regulated by inheritable differences. Although the cause of this decreased expression is still speculative, using NCBI Genome and dpSNP Database (http://www.ncbi.nlm.nih.gov/projects/SNP/snp_ref.cgi?rs=12940701), we did identify 38 single-nucleotide polymorphisms (SNPs) on chromosome 17q21 (46114527…..46114613, complement1) region where miR-152 is located. Of these, rs200114569, which is associated with a C to G nucleotide change, and rs12940701, which is associated with a C to T change, were of interest because they may affect the methylation status of the miR-152 gene. The rs12940701 SNP has a relatively high frequency (15%) of the minor T allele in the European American population (n=120) relative to the Yoruba (Nigerian) population (n=118). Thus, the European American population could have lower methylation rates since the C nucleotide is changed to the T nucleotide, whereas, in the Yoruba population, this shift is less frequent, allowing for more C nucleotides, thus possibly increasing the rate of methylation. Further analysis should focus on the methylation frequency of miR-152 and on genotyping SNPs that compromise miR-152 expression in AAs and CAs.

In summary, the present work highlights miR-152 as a tumor suppressor that is inactivated by methylation. Because a large number of tumor/metastasis suppressor genes are silenced as a result of methylation, miR-152 could be a central regulator of key events that contribute to tumorigenesis and aggressiveness and thus has the potential to be a therapeutic agent for PCa treatment. These results begin to unravel the molecular mechanism associated with the aggressive tumors of AA patients.

## MATERIALS AND METHODS

### PCa cell lines and primary tissue samples

Immortalized PCa cell lines RC-77T/E (T3c poorly differentiated primary tumor), RC-77N/E (non-malignant) were derived from AA patient as previously described [21]. RC-43T/E (T4 poorly differentiated primary tumor) and RC-43N/E (non-malignant) were also obtained from an AA patient (characterization unpublished). Benign cell lines derived RC-165N/hTERT (derived from AA patient) [33] and RC-170N/hTERT (derived from CA patient) [34], and primary tumor cell line RC-92a/hTERT (derived from CA patient) cells [35]. All cell lines were cultured in keratinocyte serum-free medium (KGM, LifeTechnologies, Carlsbad, CA) supplemented with bovine pituitary extract, recombinant epidermal growth factor, and 1% penicillin-streptomycin-neomycin were maintained in KGM medium as previously described [36]. Non-malignant CA prostate epithelial cells (PrEC) were obtained from Clonetics Lonza (Switzerland) and maintained in Prostate Epithelial Cell Growth Medium (Clonetics). The CA androgen-independent and metastatic PC-3 and DU-145 PCa cell lines were maintained in Dulbecco's Modified Eagle medium (DMEM) supplemented with 10% fetal bovine serum and 1% penicillin-streptomycin-neomycin. The CA LNCaP and C4-2b androgen-dependent and -independent, respectively, prostate cells were maintained in T-medium. RWPE-1 normal prostate cells were maintained in KGM as previously described [37]. The malignant, androgen-receptor positive AA MDA-PCa-2b cells were purchased from ATCC (Manassas, VA). These cells were maintained in F-12K medium supplemented with 20% fetal bovine serum, 25 ng/ml cholera toxin, 10 ng/ml mouse epidermal growth factor, 5 μM phosphoethanolamine, 100 pg/ml hydrocortisone, 45 nM selenious acid, and 5 μg/ml bovine insulin.

### Patient Samples

Patient samples were obtained from the Cooperative Human Tissue Network at the University of Alabama at Birmingham (UAB) under Institutional Review Board-approved protocols and the St. Frances Hospital and Medical Center, Hartford CT. Additionally, the Institutional Review Boards of Tuskegee University St. Frances, and UAB approved the use of tissues for this study. Tumor sections were macro dissected. There was no significant difference between the two groups with respect to tumor content. (Average percentages of tumor for AA and CA biopsies were both 54%.) Paired normal tissues were also collected from the same patients. Altogether, samples from each of the 20 AA and 19 CA patients, along with patient-matched normal adjacent tissues, were used for qRT-PCR validation. p-values were generated using graph pad software. T-tests (the Benjamini-Hochberg corrected for multiple tests that reflects the individual patients with significant difference in mir152 expression relative to global mean of u48 for Ct values and global mean for delta Ct values) were performed to test for significant differences between the means, and F-tests were used to assess differences in variance between experimental groups.

### Quantitative Real-time PCR (qRT-PCR)

Total RNA extraction was performed using the Ambion recover all nucleic acid isolation kit (AM 1975) modified by replacing filters with (AM10066G). RNA (10 ng) was reverse transcribed using TaqMan miRNA reverse transcription kits (Life Technologies). For mRNA expression, 1 μg of total RNA was reverse transcribed using High Capacity cDNA kits (Invitrogen). Relative expression of miRNAs and mRNA was quantified with the TaqMan Universal PCR Master Mix, No AmpErase UNG, with the 7500 Fast Real-Time PCR system (Life Technologies). Thermal cycling conditions included enzyme activation for 10 min at 95°C, 40 cycles of 95°C for 15 s, and 60°C for 60 s, according to provider's protocol. Reverse transcription for mRNA was accomplished as previously described [22, 38]. SYBR Green reagents (Invitrogen) were used for quantitative real-time PCR. Thermal cycling conditions for primer sequences used for mRNA detection are included in the table below. Analyses for miRNA and mRNA were performed in triplicate. RNU48 miRNA, GAPDH, and 18S ribosomal RNA were used as endogenous controls.

### Transfection of miRNA Precursor

LNCaP, PC-3, and MDA-PCa-2b PCa cell lines were seeded at 2 × 10^5^ cells in six-well plates on the day before transfection. Lipofectamine 2000 reagent (Life Technologies) was used to transfect 5-15 nmol of miR-152 or 5-15 nmol of miR-Negative Control #1 in Opti-MEM (Life Technologies). Cells were harvested for assays three days after recovery in fully supplemented media, as described previously [38].

### Cell Proliferation Assays

LNCaP, PC-3, and MDA-PCa-2b cells (2.5 × 102) were plated in 96-well plates in DMEM. After being cultured for 24 hr, cells were transfected with miR-152 or Negative Control #1 (Life Technologies) at a concentration of 30 nM. Cell viability was determined at 24, 48, 72, 96, and 120 days after transfection. All cells were incubated with 10 μl of 3-(4,5-dimethylthiazol-2-yl)-2,5-diphenyltetrazolium bromide (MTT, Sigma-Aldrich, St. Louis, MO) solution (5 mg/ml) in PBS for 4 hr. Dimethylsulfoxide (50 μl) was added for 10 min after aspiration of the MTT solution. Plates were read at 560 nm.

### Cell Migration and Invasion assay

Cell migration and invasion was determined using the Boyden chamber assay. Briefly, 20,000 cells were plated in the upper chamber, with or without Matrigel, containing serum-free medium containing 1% bovine serum albumin for 24 hours; this then was replaced with a serum-free medium for an additional 24 hours. The lower chamber contained medium with 10 ng/mL of epidermal growth factor was used as a chemo-attractant. The number of cells that migrated or invaded through the matrix over a 48 hour-period was determined by counting cells that stained with crystal violet on the bottom of the filter. All experiments were performed in triplicate.

### Sodium Bisulfite Modification and Methylation Analysis

DNA was extracted with GenElute Mammalian Genomic DNA Miniprep kits (Sigma-Aldrich) following the manufacturer's instructions. Subsequently, bisulfite conversion of DNA (2 μg) was performed with EpiTect Bisulfite kits (Qiagen, Valencia, CA). The miR-152 sequence was determined by using the UCSC Genome Browser [39]. Methylation PCR amplification primers for sequencing were designed using MethPrimer [40]. Sequence from 5' to 3' - Primer 1: GGGTTAGGGGGAGTAGTTAATTTAG and Primer 2: ATAAACTCCAAAAACATACCCATCA. qRT-PCR was performed on bisulfite-converted DNA (1 μl), using the primers described above covering two CpG regions upstream from the miR-152 promoter DNA sequence. A second round of amplification was performed on the first PCR amplicons and subsequently characterized by electrophoresis on 1.2% agarose gels. Sequencing of amplicons was performed at the UAB Heflin Center, Birmingham, AL. Bisulfite sequencing was analyzed by an online DNA methylation platform, Bisulfite Sequencing Data and Presentation Compilation (http://biochem.jacobs-university.de/BDPC/).

### Immunoblots

Cells were harvested 3 days after miR-152 transfection, washed with PBS, and lysed in NP-40 lysis buffer [10 mM Tris-Cl (pH 7.4), 10 mM NaCl, 3 mM MgCl_2,_ 0.5% NP-40 (Nonidet P-40), 0.15 mM spermine, 0.5 mM spermidine, and protease inhibitor solution] and centrifuged at 12,000 g for 10 min at 4°C. Protein concentrations were determined with the BCA Protein Assay kit (Thermo Scientific, Rockford, IL) according to manufacturer's instructions. Protein samples were separated on 7.5% SDS-PAGE pre-cast gels (Thermo Scientific). Protein levels were detected by anti-DNMT1, anti-mTOR, and anti-Rictor rabbit polyclonal antibodies (Cell Signaling, Danvers, MA), and analyzed by chemiluminescence.

### Treatment with 5-aza-2'-d

Cells (60% confluent) were treated with 5 μM 5-aza-2'd for 5 days, with fresh media supplemented with 5-aza-2'd every day. Trichostatin A (TSA, a histone deacetylase inhibitor) was administered at a concentration of 100 nM on the last day of treatment. Cells were harvested and assayed for miR-152 expression by qRT-PCR as previously described [21, 22].

### Flow Cytometry Analysis

Cells were harvested 3 days after miR-152 transfection, washed with cold PBS, fixed, and permeabilized with 70% cold ethanol for propidium iodide staining. Cell cycle analysis was performed with a flow cytometer (Accuri, Ann Arbor, MI).

### miRNA microarray samples

For miRNA expression profiling, 11 unique cell lines were utilized; specifically, PrEC, RC-77N/E, RC-77T/E, RC-43T/E, RC-43N/E, RC-165N/hTERT, RC-92a/hTERT, RC-170N/hTERT, PC-3, MDA-PCa-2b, and C42B. Microarray hybridizations were performed in triplicate for each line by Ausurgen, Austin TX). In addition, primary tumor samples (n=9) were also processed for microarray hybridization (RC-30T, RC-33T, RC-136T, RC-139T, RC-28T, RC-78T, RC-143T, RC-145T and RC-25T). Clinical characteristics are listed in (supplemental table 4). The protocols for RNA extraction and miRNA microarray hybridizations were performed by an outside vendor (Asuragen, Austin, TX).

### Raw data processing from microarrays

For data processing, the bioconductor packages pipelined through R interfaces DaNTE [41] and CARMA [42] were used. Pre-processing and normalization was completed with the Affy package. Two distinct array platforms were used during the course of this project; cell lines were hybridized to one platform and primary samples were hybridized to another. The platforms differ in probe content and replication. Arrays of identical platforms were quantile normalized to minimize inter-array noise [43], using the gcRMA method [44]. Subsequently, replicates were merged into median values for statistical association analyses.

### Statistical analysis of normalized data

For all analyses, p-values <0.05 were considered as significant. Initial differential expression was detected using an ANOVA linear model. Of the miRNA genes that showed significant variance, we measured the specific differential expression among categorical groupings; diagnosis, pathological stage, and race. Differential expression associated with categories was measured using a moderated T-test in the limma package of R. Cells lines representing tumor and uninvolved from the same individuals were grouped into two categories; Malignant or Non-malignant. This grouping also included established laboratory cell lines (PREC, PC3 and C42b). We incorporated multiple hypothesis testing to correct for false positive (type 1 errors) and false negative (type 2 errors). Specifically, we implemented adjusted p-values (Bonferroni) and false discovery rates (FDR) using the Benjamini and Hochberg method [45]. A moderate 10% FDR cutoff was used when applicable. We also used hierarchical clustering analyses to determine groups of differentially expressed miRNAs between specific variables and to isolate specific nodes of probes that shared similar expression trends.

We used non-parametric tests when comparing across array platforms to validate the significance of specific factors in a given variable category. Specifically, a Kruskal-Walis test was used for all variables. In addition, for variables with only 2 levels, a Wilcoxon Rank Sum test was utilized for secondary validation. In cases where strict fold change values were of importance, the fold change between two factor levels was calculated using the ratio of mean expression between the variables over differences in expression between the variables. [log(x/y) = log(x) − log(y)].

To determine the ability of significant probe profiles to differentiate samples into relevant categories, we used a partial least squares (PLS) principle component test. Specifically, once probes were found to be significantly differentially expressed, following hierarchical clustering, we identified suitable nodes or combination of probes, for each category. We determined differentially expressed probes based on diagnosis, stage and as the predictors and miRNA expression values as the response. These tests both determined whether the probes statistically associated with a specific variable or had predictive value across independent primary samples not utilized in the initial discovery ANOVA and non-parametric tests. If the largest percent variation could be explained by the variable extracted/tested, and the samples segregated according to proper category variable annotation, the probe set was considered to have significant predictive value for the specified categories. For these analyses, the DAnTE 1.2 program was utilized.

## SUPPLEMENTARY TABLES AND FIGURES


