# Supplementary material for: MicroRNA profiling of novel African American and Caucasian Prostate Cancer cell lines reveals a reciprocal regulatory relationship of miR-152 and DNA methyltranferase 1
Source: Oncotarget. 2014 May 8;5(11):3512–25. doi: 10.18632/oncotarget.1953 (PMC4116499; doi:10.18632/oncotarget.1953)
Supplement: Supplementary file 1 [file oncotarget-05-3512-s001.pdf]

MicroRNA Profiling of Novel African American and Caucasian Prostate Cancer Cell Lines Reveals a reciprocal regulatory relationship of *miR-152* and DNA methyltranferase-1

Supplementary Material

Supplemental Table 1
Differentially expressed miRNA's separated by malignancy

**Gene**	(adjusted) p-value ↑	confidence interval
**hsa-miR-520g_st2**	0.0069	(0.113, 0.662)
**hsa-miR-582_st2**	0.0121	(0.093, 0.705)
**hsa-miR-137_st2**	0.0148	(0.0776, 0.672)
**hsa-miR-501_st2**	0.0149	(-0.592, -0.068)
**hsa-miR-122a_st2**	0.0154	(-1.05, -0.119)
**hsa-miR-191_st1**	0.0171	(-0.126, -0.0132)
**hsa-miR-30a-3p_st2**	0.0175	(0.0565, 0.528)
**hsa-miR-452_st2**	0.0185	(0.0749, 0.756)
**hsa-miR-422a_st2**	0.0249	(-0.174, -0.0125)
**hsa-miR-612_st2**	0.0263	(0.0179, 0.268)
**hsa-miR-424_st2**	0.0297	(0.0467, 0.832)
**hsa-miR-452-_st2**	0.0299	(0.0646, 1.16)
**hsa-miR-154_st2**	0.0306	(0.0521, 0.972)
**hsa-miR-99a_st1**	0.0309	(-0.248, -0.0127)
**hsa-miR-224_st1**	0.032	(0.0481, 0.971)
**hsa-miR-519a_st2**	0.033	(0.0389, 0.86)
**hsa-miR-455_st2**	0.035	(0.0493, 1.25)
**hsa-miR-153_st1**	0.0379	(-0.953, -0.0292)
**hsa-miR-515-5p_st2**	0.0381	(-0.56, -0.0166)
**hsa-miR-498_st2**	0.0384	(0.00411, 0.141)
**hsa-miR-125b_st2**	0.0429	(-0.158, -0.00268)

***miRNAs are listed based significance (p-value <.05)**

Supplemental Table 2
Differentially expressed miRNA's separated by race

Gene	(adjusted) p-value ↑	confidence interval
**hsa-miR-363_st1**	3.43e-05	(0.83, 2.01)
**hsa-miR-132_st2**	0.000354	(0.232, 0.678)
**hsa-miR-376b_st2**	0.00188	(0.254, 1)
**hsa-miR-410_st2**	0.00291	(0.268, 1.18)
**hsa-miR-152_st2**	0.00471	(0.116, 0.552)
**hsa-miR-189_st2**	0.00498	(0.448, 2.17)
**hsa-miR110_st2**	0.00514	(0.168, 0.863)
**hsa-miR-27b_st2**	0.00657	(0.0455, 0.244)
**hsa-miR-519c_st2**	0.0071	(-0.635, -0.115)
**hsa-miR-520h_st2**	0.00774	(-0.684, -0.115)
**hsa-miR-27a_st1**	0.00837	(0.0425, 0.245)
**hsa-miR-382_st2**	0.00918	(0.106, 0.681)
**hsa-miR-574_st2**	0.0135	(0.0633, 0.498)
**hsa-miR199_st2**	0.0136	(0.0928, 0.745)
**hsa-miR-363-_st2**	0.0136	(0.0939, 0.746)
**hsa-miR247_st1**	0.0144	(0.0892, 0.743)
**hsa-miR-516-5p_st2**	0.0154	(0.0931, 0.807)
**hsa-miR-210_st2**	0.0156	(0.0293, 0.258)
**hsa-miR-654_st2**	0.0161	(0.0305, 0.27)
**hsa-miR155_st2**	0.0164	(-0.373, -0.0405)
**hsa-miR-499_st2**	0.0175	(0.0732, 0.697)
**hsa-miR-20a_st1**	0.0208	(0.0144, 0.161)
**hsa-miR-502_st2**	0.0211	(-1, -0.0877)
**hsa-miR-24_st2**	0.0214	(0.0176, 0.191)
**hsa-miR-631_st2**	0.0223	(-1.48, -0.131)
**hsa-miR-376a_st1**	0.0233	(0.0712, 0.905)
**hsa-miR-204_st1**	0.0236	(0.2, 2.45)
**hsa-miR109_st1**	0.0237	(0.034, 0.44)
**hsa-miR-638_st1**	0.024	(-0.0507, -0.00384)
**hsa-miR-199b_st1**	0.0242	(0.0853, 1.14)
**hsa-miR-199a-_st2**	0.0247	(0.0608, 0.832)
**hsa-miR-375_st2**	0.0261	(0.0385, 0.569)
**hsa-miR-7_st2**	0.027	(0.0205, 0.317)
**hsa-miR-520g_st2**	0.0326	(0.0348, 0.743)
**hsa-miR186_st2**	0.0326	(0.0664, 1.35)
**hsa-miR-495_st2**	0.0336	(0.0453, 1.05)
**hsa-miR197_st1**	0.0356	(-0.621, -0.0237)
**hsa-miR-17-5p_st2**	0.0358	(0.00594, 0.16)
**hsa-miR-154-_st1**	0.0385	(0.0315, 1.08)
**hsa-miR-20b_st1**	0.0402	(0.00538, 0.215)
**hsa-miR-335_st2**	0.0405	(0.0129, 0.534)
**hsa-miR-379_st2**	0.0416	(0.00758, 0.367)
**hsa-miR-365_st2**	0.0462	(0.00507, 0.547)
**hsa-miR-138_st2**	0.0466	(0.00786, 0.956)
**hsa-miR234_st1**	0.0468	(0.0031, 0.412)
**hsa-miR-17-3p_st2**	0.049	(0.000892, 0.392)
**hsa-miR236_st2**	0.0497	(0.000796, 1.05)

*miRNAs are listed based significance (p-value <.05)

Supplemental Table 3:

AA tumors	mir152.est	mir152.pval	Delta-CT.est	Delta-CT.pval
447A	-0.696637155	0.8686926	-22.0931862	0.015334445
483A	-0.57242203	0.487776836	-28.03782204	0.000135695
472A	1.230001455	0.114486832	-30.00683943	6.62E-05
587A	-1.077526095	0.8686926	-23.62551008	0.040558692
629A	0.866304395	0.373950868	-28.72112884	0.000179342
759A	5.28520203	0.060393725	-32.32330613	0.000772557
847A	3.95562363	0.337042166	-32.99103206	0.003786121
849A	5.42199135	0.002691448	-31.9771752	6.62E-05
806A	4.350014685	0.015947428	-32.48747069	0.000135695
908A	2.42042828	0.009636856	-30.89394584	6.62E-05
965A	2.718897825	0.0154044	-28.36012012	6.62E-05
687A	3.251464845	0.009636856	-28.81113801	6.62E-05
694A	3.20696258	0.001446297	-28.71584134	6.55E-05
721A	4.00127125	0.141128671	-30.13029319	0.001325822
729A	2.53715801	0.111097062	-29.78596537	0.000333294
285A	10.43512345	0.0001899	-30.71976138	6.62E-05
772A	10.31064225	0.001368501	-35.39410426	8.01E-05
757A	3.025343885	0.502017589	-31.79547242	0.006206846
779A	3.06494426	0.015334445	-34.18088515	6.62E-05
814A	4.10736084	0.007931643	-31.21567727	6.62E-05

CA tumors	mir152.est	mir152.pval	Delta-CT.est	Delta-CT.pval
518A	3.1660223	0.517993809	-28.34846599	0.010200113
490A	-1.46173382	0.628240593	-26.993535	0.003581743
507A	-2.60991573	0.713087061	-21.70785487	0.049214098
488A	4.632666585	0.002691448	-27.54621136	6.62E-05
507A-1	6.871603965	0.034732857	-27.81106009	0.001159988
813A	5.1664238	0.061944994	-27.44717286	0.001159988
901A	4.70358086	0.015289551	-27.93118704	0.0001899
864A	3.402314185	0.218540911	-30.2919182	0.001459755
903A	2.30910969	0.015334445	-27.24890009	6.62E-05
815A	5.052025795	0.045301397	-27.14304922	0.000772557
842A	2.75108528	0.011761575	-26.29559552	6.62E-05
843A	2.506352425	0.141128671	-27.09723707	0.000596418
698A	2.86456489	0.119860556	-26.50742046	0.000657323
835A	9.314155575	0.009636856	-28.52375887	0.000635486
837A	2.84062481	0.092924462	-27.3514618	0.00044229
895A	3.14495182	0.015334445	-26.33824632	9.98E-05
891A	5.56247425	0.060393725	-26.36350671	0.001368501

Supplemental Table 4:

Characteristics of African American and Caucasian Primary Tumor Cells						
Cells	Age	Race	Morphology	Pathological Stage	Tumor Grade	Gleason Score
RC-30T	65	AA	Epithelial	7	T3a	Moderate
RC-33T	56	AA	Epithelial	6	T2c	Moderate
RC-108T	70	AA	Epithelial	6	T2c	Well
RC-136T	67	AA	Epithelial	8	T2b	Poor
RC-139T	62	AA	Epithelial	9	T3c	Poor
RC-28T	55	CA	Epithelial	9	T3c	Poor
RC-143T	64	CA	Epithelial	8	T2b	Well
RC-145T	68	CA	Epithelial	7	T3b	Moderate
RC-205T	70	CA	Epithelial	8	T2b	Poor

Gene	Forward Primer	Reverse Primer
GAPDH	TGCACCACCAACTGCTTAGC	GGCATGGACTGTGGTCATGAG
SOS1	CGAGCCCTTTTCACTCAAGCAA	CAGGAGGAGGGACAGGCACTTC
Rictor	GGAAGCCTGTTGATGGTGAT	GGCAGCCTGTTTTATGGTGT
E2F1	AGATGGTTATGGTGATCAAAGCC	ATCTGAAAGTTCTCCGAAGAGTCC
DNMT1	GTGGGGGACTGTGTCTCTGT	TGAAAGCTGCATGTCCTCAC
ABCD3	CTCGGCCTGCACGGTAAGAA	TGGCAGCGATGAAGTTGAGTAAGT

Supplemental Table 5:

TGFB	AGAGTGCCTGAACAACGGATT	CCATTCGCCTTCTGCTCTT
SOX2	AACCAAGACGCTCATGAAGAAG	CTGCGAGTAGGACATGCTGTAG
SMAD4	GCTGCTGGAATTGGTGTTGATG	AGGTGTTTCTTTGATGCTCTGTCT

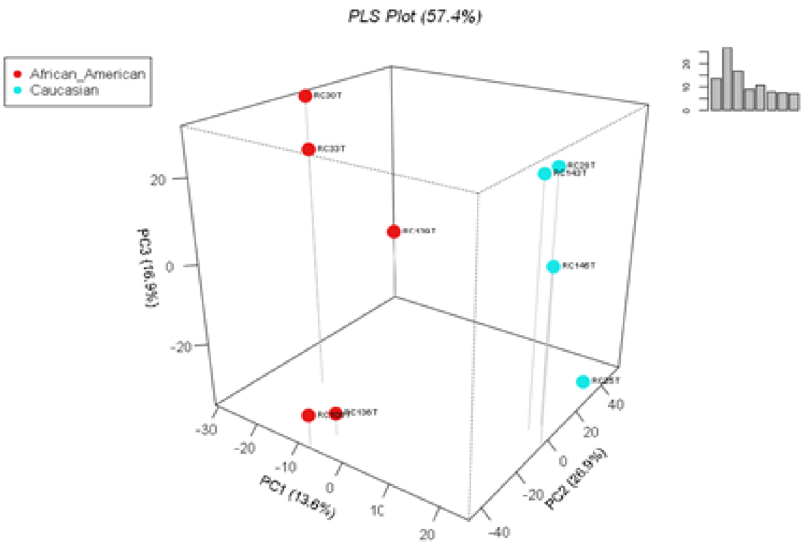

Supplemental Fig 1: **Race related miRNAs separate primary tumors cells by race.** (a) Race related miRNAs observed from the AA prostate cell lines were probed in a panel of non-immortalized AA and CA PCa cells isolated from primary tumors. PLS plot of individual primary prostate cancer cells shows distinct separation of AA and CA patients based on AA related miRNA expression.

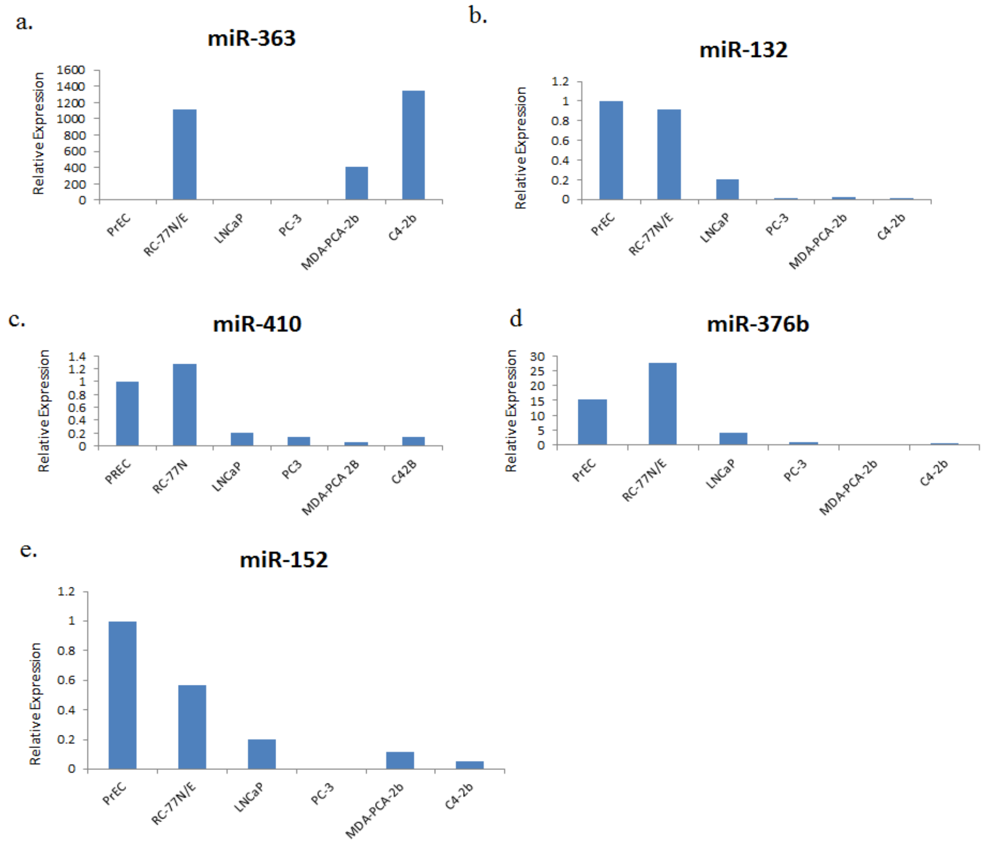

Supplemental Fig 2. **qRT-PCR validation of miRNA differentially expressed miRNA in PCa cell lines**. (a-e) Relative miRNA expression of 5 most significantly differentially expressed miRNAs were determined by qRT-PCR in prostate cancer cell lines with increasing aggressiveness, compared to normal CA PrEC and AA RC-77N/E cells. For all qRT-PCR experiments, expression was normalized to RNU48 (miRNA) control.

Location of significant microRNAs CpG Island

MicroRNA	Chromosome Location	CpG Length (bp)
**miR-152**	17q21.32	389, 203
**miR-410**	14q32.31	323
**miR-376b**	14q32.31	NA
**miR-132**	17p13.3	869

miR-152

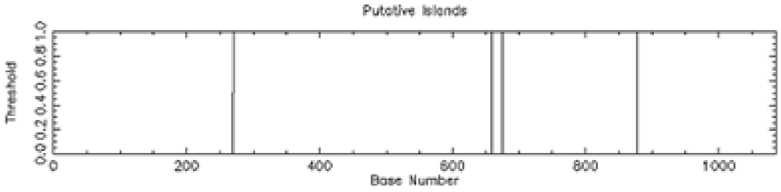

miR-410

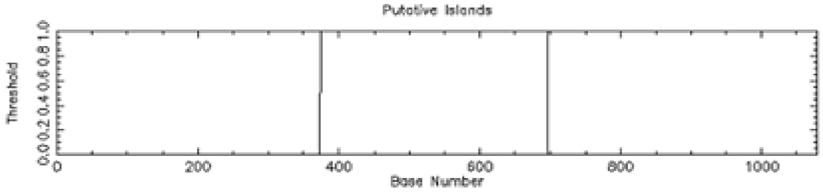

miR-376b

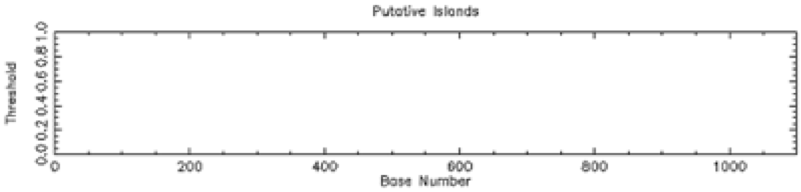

miR-132

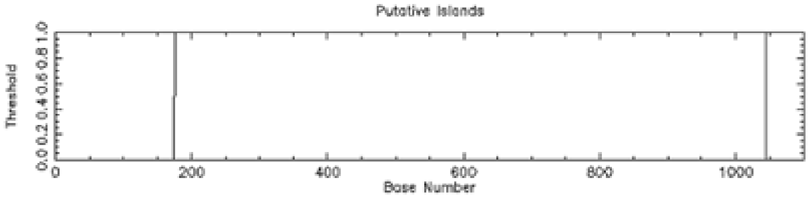

Supplemental Fig 3: ***Insilico* analysis of CpG islands of race related miRNAs.** (a). Schematic map of the CpG islands in the promoter region of miR-152, miR-410, miR-376, miR-132.

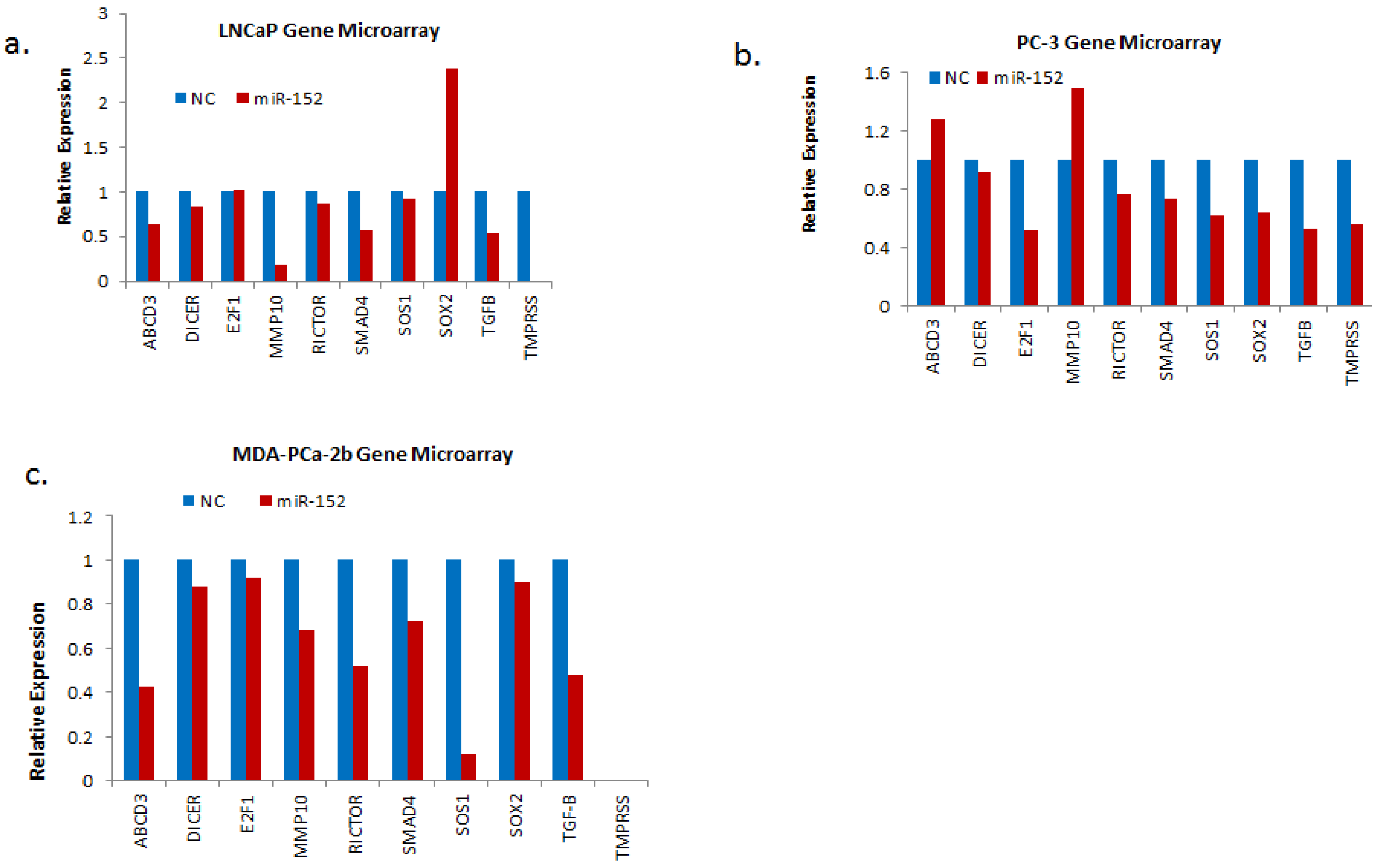

Supplemental Fig. 4: **Validation of additional miR-152 target genes** (a) qRT-PCR validation of the custom-designed miRNA microarray for alogithrim-predicted miR-152 target genes (TargetScan) with or without pre-miR-152 treatment in LNCaP, PC-3, and MDA-PCa-2b cells. For all qRT-PCR experiments, expression was normalized to GAPDH (mRNA) control.
